# Revealing the Dynamic Association Between Lymphatic Endothelial Cell Markers and Intervertebral Disk Degeneration

**DOI:** 10.3390/biomedicines14050993

**Published:** 2026-04-27

**Authors:** Qiang Zhang, Maoqiang Lin, Shishun Yan, Fei Huang, Haiyu Zhou

**Affiliations:** 1 Department of Orthopedics, Lanzhou University Second Hospital, Lanzhou 730030, China; zhqiang2023@lzu.edu.cn (Q.Z.); linmq2023@lzu.edu.cn (M.L.); yanshsh2023@lzu.edu.cn (S.Y.); huangf2024@lzu.edu.cn (F.H.); 2 Key Laboratory of Bone and Joint Disease Research of Gansu Province, Lanzhou 730030, China

**Keywords:** intervertebral disk degeneration, lymphatic vessels, matrix metabolism-related markers, dynamic changes, positive correlation

## Abstract

**Objective:** This study aims to analyze the dynamic changes in lymphatic endothelial cell (LEC) markers during the progression of intervertebral disk degeneration (IDD) and to investigate their association with the progression of IDD. **Method:** In this study, intervertebral disk (IVD) specimens were first collected from patients who underwent open lumbar fusion surgery for spinal fractures (control group, *n* = 10) and lumbar disk herniation (IDD group, *n* = 10). Concurrently, a mouse IDD model was established, and IVD specimens were collected from mouse in the Sham group and the IDD group 1, 3, and 6 weeks after modeling (*n* = 5 per group at each time point). Pathological morphological changes in human and mouse IVD specimens were observed using Hematoxylin and Eosin (H&E) and Masson’s Trichrome staining. The degree of degeneration in the mouse IVD specimens was quantified using a histopathological scoring system. Subsequently, real-time quantitative polymerase chain reaction (RT-qPCR), immunohistochemistry (IHC), and immunofluorescence (IF) staining were employed to examine LEC markers in IVD tissue, including lymphatic vessel endothelial hyaluronan receptor 1 (LYVE-1), podoplanin (PDPN), prospero homeobox protein 1 (PROX-1), and vascular endothelial growth factor receptor 3 (VEGFR-3), as well as matrix metabolism-related markers such as matrix metalloproteinase 13 (MMP-13) and collagen II (Col II). Finally, we performed Spearman’s rank correlation analysis between the histopathological scores of all mouse IVD specimens and the corresponding expression levels of LEC markers. **Results:** In human IVD tissue, expression levels of LYVE-1, PDPN, PROX-1, and VEGFR-3 were extremely low in the normal group. In contrast, expression of these markers was significantly upregulated in the IDD group. In the mouse IDD model, compared with the Sham group at the same time point, the IDD group exhibited higher histopathological scores in IVD tissue, accompanied by upregulation of LYVE-1, PDPN, PROX-1, and MMP-13, as well as downregulation of Col II. In-depth analysis revealed that these differences between the Sham and IDD groups were not static but exhibited a dynamic pattern of increasing magnitude over time. Concurrently, as the modeling period progressed, the histopathological scores of mouse IVD in the IDD group, as well as the expression levels of LYVE-1, PDPN, PROX-1, and MMP-13, showed a progressive upward trend, while Col II expression progressively decreased. In addition, Spearman’s rank correlation analysis revealed that the expression levels of LYVE-1, PDPN, and PROX-1 in mouse IVD tissue were all significantly positively correlated with histopathological scores. **Conclusions:** In the process of IDD, the dynamic upregulation of LEC markers is highly consistent with its severity in the time dimension. At the same time, there was also a significant positive correlation between the expression level of LEC markers and the severity of IDD. Taken together, these findings suggest that the dynamic upregulation of LEC markers may be potentially associated with the pathological progression of IDD.

## 1. Introduction

The intervertebral disk (IVD) is a fibrocartilaginous structure located between adjacent vertebrae, composed of the nucleus pulposus (NP), annulus fibrosus (AF), and cartilaginous endplates (CEPs) [1]. It plays a crucial role in maintaining spinal health and function. The NP occupies the central region of the IVD and is a gel-like matrix primarily composed of water, nucleus pulposus cells (NPCs), and their secreted type II collagen (Col II) and proteoglycans [2]. The AF consists of multiple concentric layers of type I collagen fibers that tightly encase the NP [3]. The CEP covers the upper and lower surfaces of the vertebral body, forming a layer of hyaline cartilage composed of chondrocytes and their secreted collagen and proteoglycans [4]. As the largest avascular tissue in the human body, the IVD relies entirely on tissue diffusion for its metabolic exchange [5]. This unique nutrient exchange mechanism, while conferring exceptional compressive strength, severely limits the tissue’s potential for self-repair and regeneration, making it more susceptible to degenerative changes [6,7].

A number of studies have shown that during the process of intervertebral disk degeneration (IDD), abnormally generated microvessels can invade the interior of IVD through AF or CEP fissures [4,5]. Although these microvessels can provide some nutritional support for IVD in the short term, they will simultaneously damage the normal structure and function of IVD, and ultimately accelerate the progress of IDD [1,2,5]. In fact, angiogenesis has been regarded as a core pathological feature of IDD, and its degree is positively correlated with the severity of IDD [3,6]. It is worth noting that in pathological conditions, lymphangiogenesis is usually secondary to angiogenesis, and the two have many similarities in molecular and cellular biology [8]. However, the distribution patterns and dynamic changes in lymphatic vessels in the IVD microenvironment have not yet been fully elucidated. Early studies found that lymphatic vessels are virtually absent in normal IVD, but their number increases significantly following degeneration [9,10,11]. Recently, Zou et al. proposed a contrary view, suggesting that lymphatic vessels are always present in normal IVD, and their number actually decreases following degeneration [12]. A thorough analysis reveals that these theoretical discrepancies may stem from the fact that the research primarily focuses on comparing static samples at a single point in time, failing to fully elucidate the dynamic evolution of lymphatic vessels during the progression of IDD.

Based on this, we hypothesize that lymphatic vessels may represent a dynamic pathological factor in the IDD process that has not yet been fully recognized. To test this hypothesis, this study first examined differences in the expression of lymphatic endothelial cell (LEC) markers between normal and degenerated IVD tissues. Subsequently, by establishing a mouse model of IDD, we dynamically analyzed the changes in histopathological scores, LEC marker expression, and matrix metabolism-related marker expression in IVD specimens at different time points (1 week, 3 weeks, and 6 weeks) post-modeling. Finally, we used Spearman’s rank correlation analysis to assess the correlation between histopathological scores and corresponding LEC marker expression levels in all mouse IVD specimens. This study aims to elucidate the dynamic relationship between LEC markers and IDD, thereby providing further evidence to explore the mechanisms underlying the role of lymphatic vessels in the pathological progression of IDD.

## 2. Materials and Methods

### 2.1. Collection of Human IVD Specimens

This study was approved by the Ethics Committee of the Second Hospital of Lanzhou University on 17 November 2025 (Approval No.: 2025A-1257). Subsequently, between 18 November 2025 and 16 January 2026, IVD specimens were collected from patients who underwent open lumbar fusion surgery for spinal fractures and lumbar disk herniation at the Department of Orthopedics II, The Second Hospital of Lanzhou University, with 10 cases in each group. Patient grouping was performed jointly by one senior-level physician and two associate senior-level physicians from our department, strictly adhering to the Pfirrmann classification system [13]. The spinal fracture group (control group) included 8 males and 2 females, aged 21–28 years, with a mean age of (24.300 ± 2.406) years. The lumbar disk herniation group (IDD group) included 6 males and 4 females, aged 37–73 years, with a mean age of (50.800 ± 9.849) years. As shown in Table 1, the mean age of patients in the IDD group was significantly higher than that of the control group (*p* < 0.001). There was no significant difference in gender distribution between the two groups (*p* = 0.628). All patients had complete preoperative imaging data (including X-rays and MRI) and signed informed consent forms. This study excluded patients with concomitant spinal infections, tumors, hypertension, diabetes, hyperuricemia, or immunosuppressive diseases. Additionally, to ensure sample independence, IVD tissue from a single segment was collected from each patient for analysis. Detailed baseline characteristics of the patients are shown in Supplementary Materials (Table S1).

### 2.2. Experimental Animals

Thirty C57BL/6 mouse (30 males), aged 8–9 weeks, weighing about 22 g, were obtained from Lanzhou Veterinary Research Institute, Chinese Academy of Sciences. It should be noted that in the initial mechanism exploration stage, in order to minimize the biological variations that may be introduced by gender factors and improve the consistency within the group, we only selected male C57BL/6 mice for the experiment. All mice were housed at a temperature of 23–25 °C, a humidity of 50%, and a routine condition with a 12 h light/dark cycle, and free access to food and water. The animal experiment design and protocol were approved by the Ethics Committee of the Second Hospital of Lanzhou University (Approval No.: D2025-583) on 1 July 2025 and followed the National Research Council’s “Laboratory Animal Care and Use Guidelines”. Between 2 July 2025 and 16 January 2026, all healthy mice without external injuries or infections were included in this study. Conversely, individuals experiencing intraoperative death, severe complications (such as paralysis or infection) within 48 h post-surgery, tissue sample damage during collection, or irreversible injury during the experiment were excluded. To ensure randomization between groups, a researcher blinded to the experiment generated a random number sequence for 30 mice using the RAND () function in Microsoft Excel. Mice were then assigned to groups based on the ascending order of their random numbers. The first 15 mice in the sequence were assigned to the Sham group, while the remaining 15 were assigned to the IDD group. Three observation time points were established (1 week, 3 weeks, and 6 weeks post-surgery), with 5 mice from each group included for subsequent testing. It should be noted that the sample size design in this study referenced conventional sample sizes used in previous mouse IDD model studies (*n* = 5–7/group) and the National Center for the Replacement, Reduction, and Refinement of Animals (NC3Rs) recommendations for biological replicates in rodent phenotypic studies (minimum = 5/group). This sample size (*n* = 5/group) provides the minimum statistical power required to assess biological inter-individual variability while accommodating potential experimental losses.

### 2.3. Establishment of Mouse IDD Model

This study employed percutaneous acupuncture to establish a mouse model of IDD. Due to its high reproducibility and ability to reliably replicate the key pathological features of human IDD, this model has been widely used in research on the mechanisms of IDD [14]. Prior to performing aseptic surgery to induce mouse IDD, anesthesia was administered via intraperitoneal injection of 2% sodium pentobarbital (0.2 mL/100 g). Mouse under adequate anesthesia were placed in a prone position, and the tail surgical site underwent routine disinfection. The target caudal vertebral space was located via palpation. A 25-gauge needle was vertically inserted approximately 1.5 mm into the target IVD, rotated 180° in situ, and held for 30 s to induce IDD [14]. Sham-operated mice underwent identical anesthesia, positioning, and skin disinfection. The needle was vertically inserted into the skin surface corresponding to the target IVD and held for 30 s without any puncture or rotation of the IVD. The surgical order for all mice was determined by random draw. Postoperatively, mice were immediately returned to their housing cages and maintained under standard conditions. To eliminate potential effects of housing location or environmental factors on experimental outcomes, cages for the Sham and IDD groups were alternately arranged on the housing rack. The entire rack was rotated 180 degrees every Wednesday and Saturday. Experimental endpoints were set at 1, 3, and 6 weeks post-surgery. At these time points, tail IVD tissue was collected from mice in both the Sham and IDD groups for subsequent experimental analysis. It should be noted that mice reaching the experimental endpoint were euthanized. The procedure involved inducing deep anesthesia with 5% isoflurane gas (confirmed by the absence of pain reflexes via the tail pinch test), followed by cervical dislocation at the atlanto-occipital joint.

### 2.4. Histological Analysis

Tissue samples were embedded in paraffin blocks and sectioned at 4 μm thickness. Human and mouse IVD specimens were stained using Hematoxylin and Eosin (H&E) and Masson’s Trichrome stains, then photographed under an optical microscope. The degree of IVD degeneration was graded according to a previously described scoring system [15]. Each image was independently scored by two raters blinded to the experimental conditions, and the mean score was used for analysis.

### 2.5. Immunohistochemistry (IHC) Staining

IHC staining of human IVD tissue sections was performed using the ready-to-use UltraSensitive^TM^ SP Immunohistochemistry Kit (Catalog No.: KIT-9720; Maxin Bio, Fuzhou, China). The main steps included baking, dewaxing, dehydration, antigen retrieval, endogenous peroxidase blocking, primary antibody incubation, secondary antibody incubation, and DAB staining. To minimize experimental variability, all tissue processing, staining, and image acquisition were performed simultaneously under identical conditions using samples from the same batch. At least three non-overlapping representative fields of view were randomly selected from each section, and images were captured using an upright fluorescence microscope (Olympus, Tokyo, Japan). Quantitative analysis of the images was performed using ImageJ software (version 1.53, National Institutes of Health, Bethesda, MD, USA). All images were converted to 8-bit grayscale images prior to analysis, and a fixed threshold was applied to distinguish positive staining from the background. The percentage of positive cells was calculated by measuring the ratio of the area of positively stained pixels to the total nuclear area in each field of view. Detailed information on the antibodies used (including LYVE-1, PDPN, and VEGFR-3) is provided in Supplementary Materials (Table S2).

### 2.6. Immunofluorescence (IF) Staining

After human and mouse IVD tissue sections were baked, dewaxed, dehydrated, and antigen-recovered, they were blocked at room temperature for 30 min with a blocking solution containing 5% goat serum to prevent nonspecific binding. Subsequently, the sections were transferred to a humidified chamber and incubated with the primary antibody overnight at 4 °C. The following day, after thorough washing with PBS, the sections were incubated with CoraLite 488-labeled secondary antibody at room temperature in the dark for 1 h, and cell nuclei were counterstained with DAPI. Finally, fluorescence images were acquired using a Panoramic Histocyte Quantitative Microscope (TissueGnostics Asia Pacific Limited, Vienna, Austria). To minimize experimental variability, all tissue processing, staining, and image acquisition were performed simultaneously using samples from the same batch under identical conditions. Quantitative analysis of the images was performed using ImageJ software (National Institutes of Health, Bethesda, MD, USA). All section images were converted to 8-bit grayscale images prior to analysis, and background subtraction was performed using a fixed threshold. During analysis, cell nuclei were first delineated as regions of interest (ROIs) based on the DAPI channel, and the average fluorescence intensity of these ROIs was then measured in the corresponding fluorescence channels. Results are expressed in arbitrary fluorescence units (A.U.). Detailed information on the antibodies used (including LYVE-1, PDPN, PROX-1, COL II, and MMP-13) is provided in Supplementary Materials (Table S2).

### 2.7. Real-Time Quantitative Polymerase Chain Reaction (RT-qPCR)

Total RNA was extracted from normal and degenerated IVD tissues using the GOONIEBIO kit, and its concentration and purity were determined using a micro-volume UV spectrophotometer (DENOVIX, Wilmington, DE). Subsequently, 1 μg of total RNA was reverse-transcribed into cDNA using the PrimeScript RT kit (RR037A, TaKaRa, Otsu, Japan). Real-time quantitative PCR was performed on the ABI 7900HT Rapid Real-Time PCR System (Applied Biosystems, Waltham, MA, USA). Reactions were performed using the SYBR^®^ Green Fluorescent Quantification Kit in a total volume of 20 μL, containing 10 μL of 2× SYBR Green Pro Taq HS Premix, 0.4 μL each of forward and reverse primers, and less than 100 ng of cDNA template, with the volume made up to 20 μL with RNase-free water. The PCR amplification program was as follows: 30 s of pre-denaturation at 95 °C, followed by 40 cycles of 5 s of denaturation at 95 °C and 30 s of annealing/extension at 60 °C. Upon completion of all cycles, the temperature was immediately raised slowly from 60 °C to 95 °C, and changes in the fluorescence signal were continuously monitored to confirm the specificity of the amplification products. Gene expression levels were calculated using the 2^(−ΔΔCt)^ method with GAPDH as the internal control. The primer sequences used (including GAPDH, LYVE-1, VEGFR-3, and PROX1) are detailed in Supplementary Materials (Table S3).

### 2.8. Statistical Analysis

In this study, human data were derived from 10 independent biological replicates (*n* = 10); animal data were derived from 5 independent biological replicates (*n* = 5). To ensure the objectivity of the analysis, all statistical analyses were performed independently by two researchers who were blinded to the experimental details using GraphPad Prism 10.1.2 software (GraphPad Software Inc., San Diego, CA, USA). In the human study section, age differences between the two patient groups were analyzed using the nonparametric Mann–Whitney U test; gender differences between the two patient groups were analyzed using Fisher’s exact test. For human IVD sample data, the Shapiro–Wilk test is used to assess normality prior to analysis (α = 0.05), and Levene’s test is used to assess homogeneity of variance (α = 0.05). When data were normally distributed and homogeneous in variance, an unpaired *t*-test was used for between-group comparisons. For mouse experimental data, a two-way ANOVA was used to assess the main effects of the “group” and “time” factors on the observed measures, as well as the interaction between them. Prior to analysis, the Shapiro–Wilk test and Levene’s test were used to assess the normality and homogeneity of variance of the data. If the interaction was significant (*p* < 0.05), simple effects analysis was performed. Within-group comparisons across different time points within the same group were conducted using Tukey’s multiple comparison test; between-group comparisons across different groups at the same time point were conducted using Sidak’s post hoc multiple comparison test. It should be noted that all *p*-values reported for post hoc tests involving multiple comparisons in this study have been corrected using the Tukey or Sidak method. Additionally, Spearman’s rank correlation analysis was used to assess the strength of the association between the histopathological scores of all mouse IVD samples and the corresponding fluorescence intensity of LEC markers. The strength of the correlation was expressed as the Spearman correlation coefficient (r). Statistical significance was set at *p* < 0.05.

## 3. Results

### 3.1. LEC Markers in Human IVD Tissue

**Figure 1:**
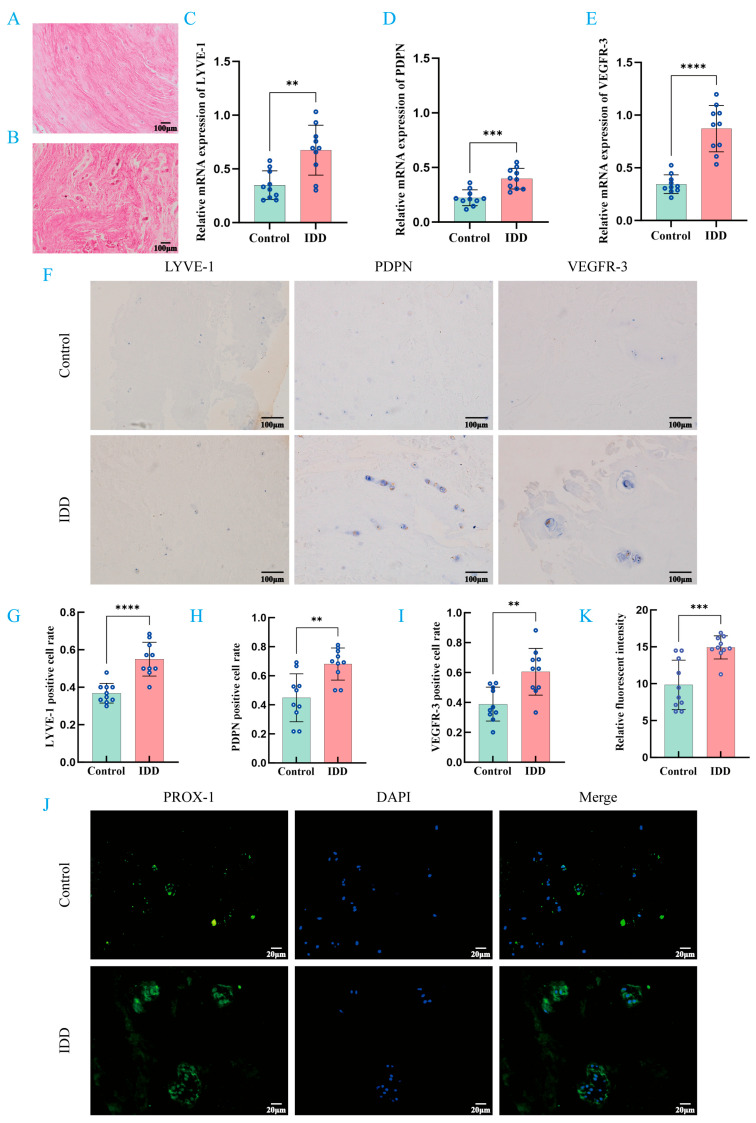
LEC markers in human IVD tissue. (**A**) H&E-stained image of IVD from a patient with spinal fracture. (**B**) H&E-stained image of an IVD from a patient with lumbar disk herniation. (**C**–**E**) PCR analysis of LYVE-1, PDPN, and VEGFR-3 in two groups of IVD sections. (**F**) Representative IHC images of LYVE-1, PDPN, and VEGFR-3 in IVD sections from both groups. Brown staining indicates a positive signal for the target protein, whilst blue indicates cell nuclei counterstained with hematoxylin. (**G**) Quantitative analysis of LYVE-1 in IVD sections from both groups. (**H**) Quantitative analysis of PDPN in IVD sections from both groups. (**I**) Quantitative analysis of VEGFR-3 in IVD sections from both groups. (**J**) Representative IF images of PROX-1 in IVD sections from both groups. (**K**) Quantitative analysis of PROX-1 in IVD sections from both groups. ** *p* < 0.01, *** *p* < 0.001, **** *p* < 0.0001 compared to control group. Values are presented as means ± SD from 10 independent experiments. For specific data, please refer to Supplementary Materials (Table S4).

Specimens of IVD collected from patients with spinal fractures were included in the control group (normal group), while specimens collected from patients with lumbar disk herniation were included in the IDD group. The results of H&E staining showed that NPC was scattered in the control group, the extracellular matrix showed a uniform and loose network structure, and the collagen fibers were arranged in an orderly manner (Figure 1A,B). In contrast, the IVD tissue of the IDD group showed obvious signs of degeneration. Its characteristics included the condensation of NPC and extracellular matrix into clusters, and the disordered arrangement of collagen fiber rings (Figure 1A,B). At the same time, in order to further clarify the distribution characteristics of LEC markers in human IVD tissues, we also compared the expression differences in LEC markers (LYVE-1, PDPN, PROX-1 and VEGFR-3) between the two groups of IVD specimens. The results of PCR showed that the mRNA expression levels of LYVE-1, PDPN and VEGFR-3 in degenerative IVD tissues were significantly higher than those in the control group (Figure 1C–E). IHC staining obtained the same results. In the control group, no positive signals of LYVE-1, PDPN and VEGFR-3 were detected (Figure 1F–I). In contrast, in degenerative IVD tissues, the number of positive cells for these markers was significantly increased (Figure 1F–I). In addition, IF staining showed that the green fluorescence signal of PROX-1 in degenerative IVD tissues was also significantly enhanced (Figure 1J–K). Based on the above results, the LEC markers in the normal group were only slightly expressed, while the expression level in the IDD group was significantly increased.

### 3.2. LEC Markers in Mouse IVD Tissue

**Figure 2:**
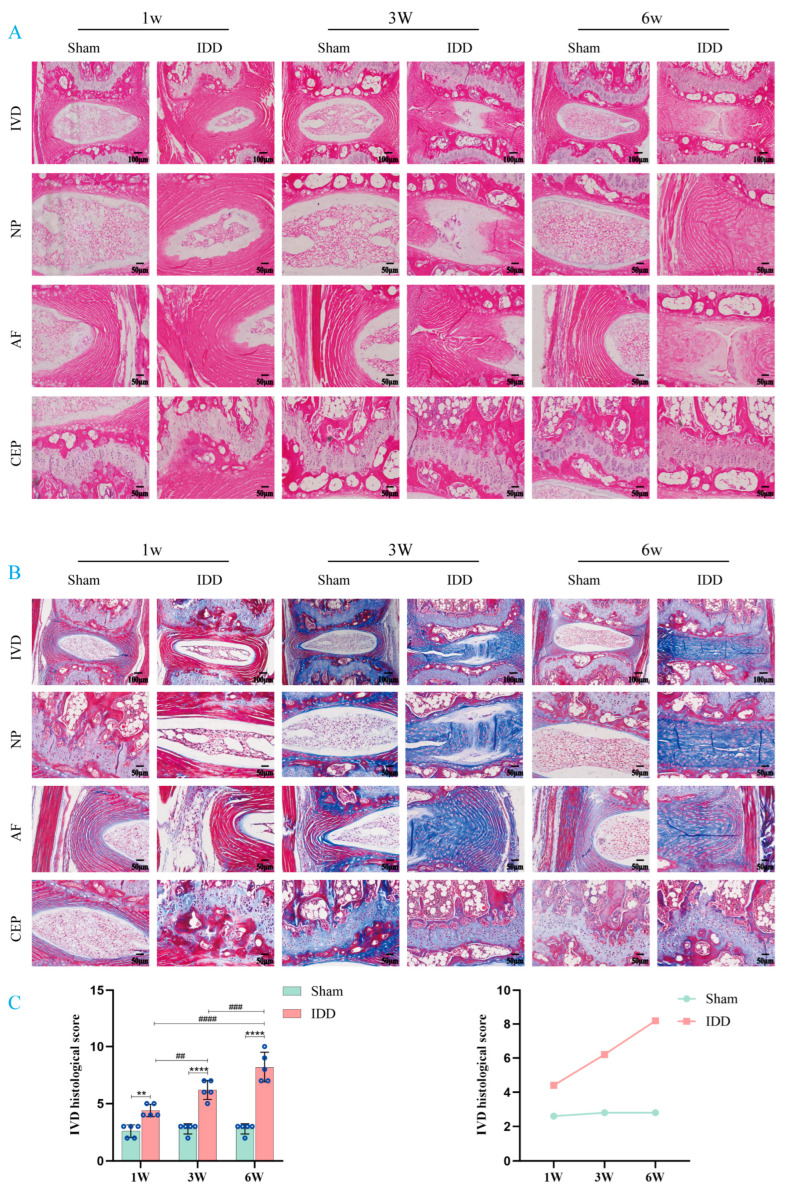
LEC markers in mouse IVD tissue. (**A**) H&E-stained image of mouse IVD. (**B**) Masson-stained image of mouse IVD. (**C**) Histopathological score of mouse IVD. ** *p* < 0.01, **** *p* < 0.0001 compared to Sham group; ^##^ *p* < 0.01, ^###^ *p* < 0.001, ^####^ *p* < 0.0001 compared to IDD group. Values are presented as means ± SD from 5 independent experiments. For specific data, please refer to Supplementary Materials (Tables S5–S8). Note: In this study, no significant differences were observed between the Sham group at different time points (1 week, 3 weeks, 6 weeks).

We first examined the microscopic structural changes in mouse IVD tissue at different time points (1 w, 3 w, and 6 w) following modeling using H&E and Masson’s staining (Figure 2A,B). The results showed that the IVDs of mice in the Sham group maintained normal morphological structure at all time points, with no obvious signs of degeneration. In contrast, the IVDs of mice in the IDD group exhibited varying degrees of degenerative changes at all time points. Characteristic features included a reduction in the area of the NP accompanied by fibrosis, disorganization, laxity, or rupture of the AF, and a decrease in overall IVD height. Based on these staining results, we performed histopathological scoring to assess the degree of IVD degeneration (Figure 2C). Statistical analysis revealed that “group,” “time,” and the “interaction” between the two had significant effects on the histopathological scores (all *p* < 0.0001). At each time point, the histopathological scores in the IDD group were significantly higher than those in the Sham group at the corresponding time points. Furthermore, the difference between the two groups widened significantly over time, and the scores within the IDD group exhibited a significant progressive increase.

**Figure 3:**
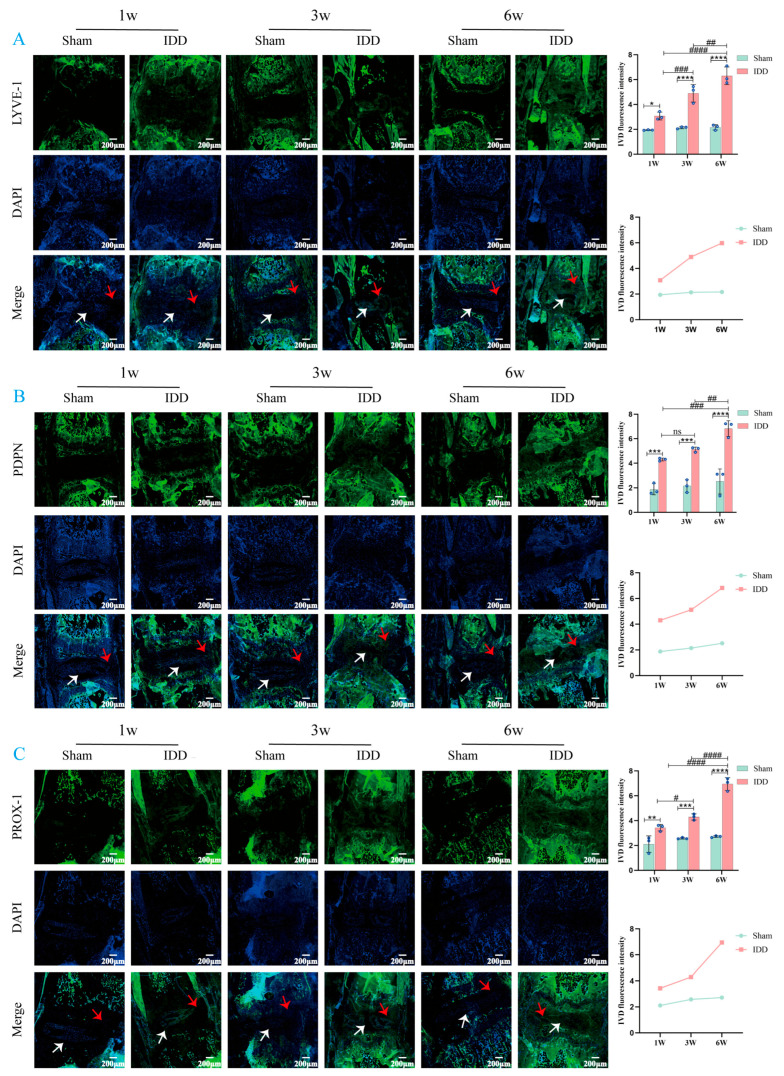
LEC markers in mouse IVD tissue. (**A**) Representative IF image and quantitative analysis of LYVE-1 in mouse IVD sections. (**B**) Representative IF image and quantitative analysis of PDPN in mouse IVD sections. (**C**) Representative IF image and quantitative analysis of PROX-1 in mouse IVD sections. The white arrow indicates the NP region, and the red arrow indicates the AF region. * *p* < 0.05, ** *p* < 0.01, *** *p* < 0.001, **** *p* < 0.0001 compared to Sham group; ^#^ *p* < 0.05, ^##^ *p* < 0.01, ^###^ *p* < 0.001, ^####^ *p* < 0.0001, ns (*p* > 0.05) compared to IDD group. Values are presented as means ± SD from 3 independent experiments. It should be noted that, due to technical limitations during the quantitative fluorescence analysis, samples from two mice each in the Sham and IDD groups were excluded at weeks 1, 3, and 6 post-surgery (this exclusion was performed independently by the quantitative analysis personnel). For specific data, please refer to Supplementary Materials (Tables S5–S8). Note: In this study, no significant differences were observed between the Sham group at different time points (1 week, 3 weeks, 6 weeks).

**Figure 4:**
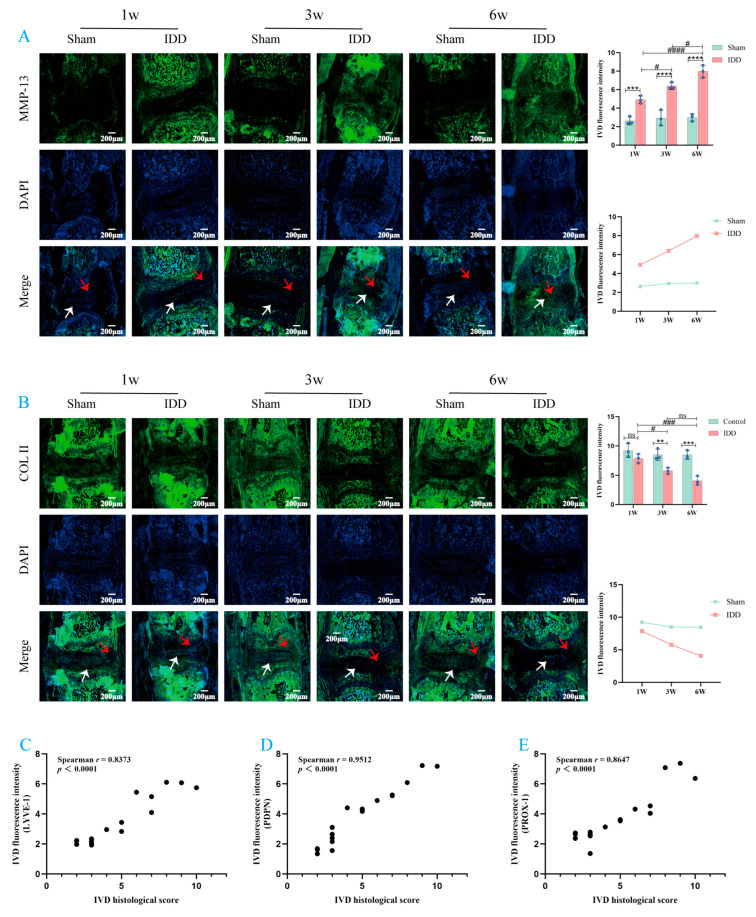
(**A**) Representative IF image and quantitative analysis of MMP-13 in mouse IVD sections. (**B**) Representative IF image and quantitative analysis of Col II in mouse IVD sections. (**C**) Spearman’s rank correlation analysis of LYVE-1 fluorescence intensity and histopathological scores in mouse IVD sections. (**D**) Spearman’s rank correlation analysis of PDPN fluorescence intensity and histopathological scores in mouse IVD sections. (**E**) Spearman’s rank correlation analysis of PROX-1 fluorescence intensity and histopathological scores in mouse IVD sections. The white arrow indicates the NP region, and the red arrow indicates the AF region. ** *p* < 0.01, *** *p* < 0.001, **** *p* < 0.0001, ns (*p* > 0.05) compared to Sham group; ^#^ *p* < 0.05, ^###^ *p* < 0.001, ^####^ *p* < 0.0001, ns (*p* > 0.05) compared to IDD group. Values are presented as means ± SD from 3 independent experiments. It should be noted that, due to technical limitations during the quantitative fluorescence analysis, samples from two mice each in the Sham and IDD groups were excluded at weeks 1, 3, and 6 post-surgery (this exclusion was performed independently by the quantitative analysis personnel). For specific data, please refer to Supplementary Materials (Tables S5–S8). Note: In this study, no significant differences were observed between the Sham group at different time points (1 week, 3 weeks, 6 weeks).

Subsequently, to further investigate the dynamic relationship between LEC markers and IDD, we examined changes in the expression of LEC markers (LYVE-1, PDPN, PROX-1) and matrix metabolism-related markers (MMP-13, Col II) in mouse IVD tissue at various time points using immunofluorescence (IF) staining (Figure 3A–C, Figure 4A,B). The results showed that, compared with the Sham group at the same time points, the expression of LYVE-1, PDPN, PROX-1, and MMP-13 was significantly elevated in the IDD group, while the expression of Col II was significantly decreased (except at week 1). Further analysis revealed that in the IDD group, the expression of LYVE-1, PDPN, PROX-1, and MMP-13 progressively increased over time, while Col II expression progressively decreased. In fact, the expression of LYVE-1, PDPN, PROX-1, MMP-13, and Col II was not only significantly influenced by both “group” (all *p* < 0.0001) and “time” factors (*p*-values of <0.0001, 0.0015, <0.0001, 0.0007, 0.0019), but also exhibited significant “interactions” (*p* = 0.0003, *p* = 0.0383, *p* < 0.0001, *p* = 0.0041, *p* = 0.0297). Specifically, the differences in the expression of all the aforementioned markers between the Sham group and the IDD group were not constant but showed a trend of dynamically widening over time. Furthermore, it should be noted that during this dynamic process, the expression of some markers exhibited slight fluctuations at specific stages. For example, there was no significant difference in PDPN expression in the IDD group between week 1 and week 3; similarly, no statistical difference was observed in Col II expression between week 3 and week 6. In summary, the dynamic upregulation of LEC markers in IVD tissue exhibits a synchronous evolution with the severity of IDD.

Finally, to clarify the relationship between LEC marker expression levels and the severity of IDD, we performed Spearman’s rank correlation analysis to assess the correlation between the histopathological scores of all mouse IVD specimens and the corresponding LEC marker expression levels (Figure 4C–E). The results revealed that the expression levels of LYVE-1, PDPN, and PROX-1 in IVD tissue were significantly positively correlated with histopathological scores. These findings suggest that the expression of LEC markers may play a significant role in the pathological progression of IDD.

## 4. Discussion

To date, there is no consensus within the academic community regarding the distribution patterns and dynamic changes in lymphatic vessels in the IVD microenvironment. The traditional view holds that an initial lymphatic network is present in the embryonic AF, but these structures typically undergo complete regression by age 4–11 [10,11]. However, recent studies have demonstrated detectable lymphatic structures in AF from young individuals (≤20 years old), with evidence suggesting that lymphatic vessels persist throughout the adult IVD [11,12]. Furthermore, conflicting reports exist regarding the number of lymphatic vessels in normal versus degenerative IVDs [9,12,16]. It is worth noting that the above conclusions are primarily based on static observations of LEC markers in IVD tissues at specific stages, while data on their dynamic changes during the IDD process remain relatively scarce. To address this research gap, we conducted dynamic observations and analyses of changes in LEC markers during the progression of IDD by integrating human samples with mouse models. In human IVD tissue, the expression levels of LEC markers were extremely low in the normal group, whereas they were significantly elevated in the IDD group. In the mouse IDD model, there were significant differences between the Sham and IDD groups in terms of histopathological scores, LEC marker expression, and the expression of markers related to matrix metabolism. Statistical analysis further confirmed that these differences not only exhibited significant “group” and “time” effects but also demonstrated a significant interaction.

Specifically, in the IDD group, the histopathological scores of the IVD and the expression levels of LYVE-1, PDPN, PROX-1, and MMP-13 were all significantly higher than those in the Sham group at the same time point, while Col II expression was significantly reduced. Further observation of the IDD group revealed that, as the duration of the model increased, the histopathological scores of the IVD and the expression levels of LEC markers and MMP-13 showed a progressive upward trend; conversely, Col II expression exhibited a progressive decline. Crucially, these intergroup differences were not static but followed a time-dependent pattern of dynamic expansion. Taken together, these results indicate that the dynamic upregulation of LEC markers evolves in tandem with the progression of IDD. This core finding is strongly supported by Spearman’s correlation analysis. In mouse IVD tissue, the expression levels of LYVE-1, PDPN, and PROX-1 all showed significant positive correlations with histopathological scores. Taken together, these findings suggest that the dynamic upregulation of LEC markers may be potentially associated with the pathological progression of IDD.

So, what role do lymphatic vessels actually play in the progression of IDD? This is a key question that remains unanswered in the current study and represents a central focus for future research. In the following sections, we will draw on the existing literature to discuss the potential biological functions of lymphatic vessels in the progression of IDD, with the aim of advancing further exploration in this field. Previous studies have suggested that the absence of lymphatic vessels in degenerated IVDs may lead to the accumulation of inflammatory cytokines, ECM-degrading enzymes, and growth factors in the local microenvironment, thereby accelerating the progression of IDD [9,10]. This academic perspective has received some support in recent research. Zou et al. demonstrated in a mouse model that lymphatic vessels may delay the IDD process by mediating the clearance of inflammatory cells [12]. However, it should be noted that, as this conclusion still lacks systematic experimental evidence, it has not yet gained widespread consensus within the academic community. Furthermore, research by Fu et al. indicates that promoting the formation of paravertebral lymphatic vessels can effectively reduce inflammatory responses in the IVD and delay the progression of IDD [16]. It is worth noting that the focus of this study was primarily on the paravertebral lymphatic system; whether its function corresponds to that of lymphatic vessels within the IVD tissue still requires rigorous analysis.

This study also has certain limitations. Given the significant ethical and practical challenges associated with obtaining IVD tissue from healthy individuals, we used IVD specimens from patients with Pfirrmann Grade I spinal fractures as the control group. It should be noted that such tissue is not strictly speaking normal IVD. This is because traumatic stress or underlying acute inflammation may interfere with the expression levels of LEC markers in this tissue, preventing it from fully reflecting the physiological baseline state of normal IVD. Nevertheless, given that the time interval between injury and sample collection is typically short, we believe that the expression levels of LEC markers in these samples still reflect a near-physiological state to the greatest extent possible. It is important to note that non-LEC types may also express the LEC markers examined in this study. In fact, to mitigate potential off-target risks, this study employed a strategy of co-expression of multiple markers for cross-validation. First, these markers exhibited consistent expression patterns in target cells, significantly reducing the likelihood of interference from non-target cells. Second, the intensity of marker expression also showed a dose-dependent relationship with the severity of IDD. Notably, this systematic gradient pattern differs fundamentally from the discrete distribution patterns typically observed with nonspecific signals. Collectively, these analyses indicate that the changes in marker expression detected in this study genuinely reflect the biological behavior of LECs, rather than artifacts introduced by experimental techniques. Furthermore, the current sample size of the human cohort remains limited and warrants expansion in future studies.

We fully recognize that there is a significant age difference between the control group and the IDD group. Aging itself is a complex biological process that may influence the expression of LEC markers in the IVD microenvironment through multiple mechanisms. First, the chronic low-grade inflammatory state associated with aging may indirectly promote LEC activation by inducing the expression of various inflammatory cytokines and lymphangiogenic factors. Second, the reduced remodeling capacity of aged IVD tissue leads to alterations in its matrix composition, thereby creating a microenvironment conducive to LEC migration and lymphangiogenesis. Furthermore, the formation of intravascular structures within the IVD during the aging process increases local oxygen concentration, which in turn triggers oxygen-dependent degradation of hypoxia-inducible factor-1α (HIF-1α) [17]. It is worth noting that in tumor research, HIF-1α has been shown to promote lymphangiogenesis by activating the VEGF-C/VEGFR-3 signaling axis [18]. However, it should be noted that the stratification of this study is mainly based on the severity of IDD imaging (Pfirrmann grade) rather than age. In fact, in the IDD group, the upregulation of LEC markers is far beyond the range that physiological aging can explain. More importantly, in the age-matched mouse IDD model, the trend of LEC markers we observed is highly consistent with human data, which greatly enhances the credibility of human research conclusions. In summary, we tend to believe that the pathological process of IDD itself may be the main factor driving the upregulation of LEC markers, and age is more likely to play an indirect role by accelerating the progression of IDD. Nevertheless, when interpreting the differences in the expression of LEC markers observed in human data, it is still necessary to carefully consider age differences as an important confounding factor. In addition, there may also be potential inherent biological differences between the different lumbar segments included in the study, which also constitutes a source of variation that cannot be ignored.

Although the mouse caudal spine puncture model effectively simulates the core pathological processes of IDD, it has inherent limitations. First, the fundamental differences in anatomical structure and biomechanical environment between species are difficult to overcome. The mouse caudal spine is a relatively simple biomechanical structure and cannot fully replicate the true mechanical environment of human lumbar IVDs subjected to long-term, high-intensity, multidirectional, and complex forces. Second, this model primarily replicates the pathological process following acute mechanical injury, which differs from the multifactorial, long-term cumulative chronic degeneration in humans in terms of disease progression and systemic context. Furthermore, this study was conducted exclusively in male animals and did not examine the influence of gender on IDD progression or the expression of potential LEC biomarkers. This is an area worthy of attention but has not yet been fully explored. Finally, it is important to emphasize that the findings from this model require further validation in chronic degeneration models or human samples.

## 5. Conclusions

This study not only described the distribution characteristics of LEC markers in human IVD tissues, but also found that they showed progressive upregulation in the process of IDD. More importantly, there is a significant positive correlation between the expression level of LEC markers and the severity of IDD. Based on the above evidence, the dynamic upregulation of LEC markers may be potentially associated with the pathological progression of IDD. This discovery not only helps to understand the role of lymphatic vessels in the pathological process of IDD, but also provides an important research entry point for exploring the molecular mechanism of IDD.

## Figures and Tables

**Table 1:** Demographic characteristics of the two groups of patients (*n* = 10).

Characteristic	Spinal Fracture Group	Lumbar Disk Herniation Group	*p*-Value
Age, years			<0.001
Median, IQR	24.5, (22.8–26.3)	45.5, (39.0–59.8)	
Range	21–28	37–73	
Sex			0.628
Male, *n* (%)	8 (80.0)	6 (60.0)	
Female, *n* (%)	2 (20.0)	4 (40.0)	

## Data Availability

The datasets used during the current study are available from the corresponding author upon request.

## Supplementary Materials

The following supporting information can be downloaded at https://www.mdpi.com/article/10.3390/biomedicines14050993/s1, Table S1. Information of patients. Table S2. Information for primary and secondary antibodies. Table S3. The list of primer sequences. Table S4. Statistical Analysis of the Control Group and the IDD Group (*n* = 10). Table S5. Statistical analysis data for each IDD group (mean ± SD). Table S6. Effect sizes of each factor (Partial η^2^). Table S7. Statistical Analysis of the Sham Group and the IDD Group. Table S8. Statistical Analysis of the IDD Group.
