# Supplementary material for: Revealing the Dynamic Association Between Lymphatic Endothelial Cell Markers and Intervertebral Disk Degeneration
Source: Biomedicines. 2026 Apr 27;14(5):993. doi: 10.3390/biomedicines14050993 (PMC13203476; doi:10.3390/biomedicines14050993)
Supplement: Supplementary file 1 [file biomedicines-14-00993-s001.zip › biomedicines-4248957-supplementary.pdf]

This study collected lumbar disc specimens from two patient groups at the Department of Orthopedics, Lanzhou University Second Hospital from November 2025 to January 2026.

(1) Spinal Fracture Group (10 cases): 8 males and 2 females, aged 21–28 years, with a mean age of (24.300 ± 2.406) years;

(2) Lumbar disc herniation group (10 cases): 6 males and 4 females, aged 37–73 years, with a mean age of (50.800 ± 9.849) years.

Exclusion criteria encompassed patients with spinal infections, tumors, hypertension, diabetes, hyperuricemia, and immunosuppressive disorders. Additionally, only single-segment disc tissue was harvested from all patients for inclusion in the study. Detailed baseline patient characteristics are presented in Table S1.

Table S1. Information of patients

Diagnosis	Number	Gender	Age	Segment	Pfirrmann grade
**Spinal fracture (CTR)**	1	Male	21	L1-L2	I
	2	Male	28	L1-L2	I
	3	Male	25	L2-L3	I
	4	Female	26	L4-L5	I
	5	Male	23	L1-L2	I
	6	Female	27	L2-L3	I
	7	Male	22	L3-L4	I
	8	Male	22	L4-L5	I
	9	Male	26	L2-L3	I
	10	Male	23	L4-L5	I
**Lumbar disc herniation (IDD)**	11	Male	44	L4-L5	IV
	12	Female	45	L4-L5	V
	13	Male	60	L4-L5	V
	14	Female	39	L4-L5	IV
	15	Male	73	L3-L4	V
	16	Male	66	L3-L4	V
	17	Male	46	L4-L5	IV
	18	Female	59	L4-L5	IV
	19	Male	37	L4-L5	IV
	20	Female	39	L3-L4	IV

Table S2. Information for primary and secondary antibodies

Target	Host	Company	Cat. No.	Dilution ratio
**LYVE-1**	Rabbit	HUABIO, Boston, MA, USA	ET1702-29	1: 200 for IHC
**LYVE-1**	Rabbit	HUABIO, Boston, MA, USA	ET1702-29	1: 300 for IF
**PDPN**	Rabbit	Proteintech, Rosemont, IL, USA	11629-1-AP	1: 200 for IHC
**PDPN**	Rabbit	Proteintech, Rosemont, IL, USA	11629-1-AP	1:300 for IF
**PROX-1**	Rabbit	Proteintech, Rosemont, IL, USA	11067-2-AP	1: 300 for IF
**MMP13**	Rabbit	Proteintech, Rosemont, IL, USA	18165-1-AP	1: 300 for IF
**Col II**	Rabbit	Proteintech, Rosemont, IL, USA	28459-1-AP	1: 500 for IF
**CoraLite488-conjugated Goat Anti-Rabbit IgG(H+L)**	Goat	Proteintech, Rosemont, IL, USA	SA00013-2	1: 50

Table S3. The list of primer sequences

Gene	Forward primer (5'->3')	Reverse primer (5'->3')
GAPDH	CAAATTCCATGGCACCGTCA	AGCATCGCCCCACTTGATTT
LYVE-1	GCGAACCAGCAGCTGAATTT	AACCCAGCCATAGCTGCAAG
PDPN	ACCAGTCACTCCACGGAGAA	GCGAGTACCTTCCCGACATT
VEGFR-3	GGCCGCCAGGTATTACAACT	TGTCTGGTTGTCCACAGAGC

1. Human Data

The table below lists the exact p-values for the differences between the Control group and the IDD group.

Table S4. Statistical Analysis of the Control Group and the IDD Group (n=10)

Index	P-value	95% CI of diff	Cohen's d
**LYVE-1**	*P*=0.0012 (PCR)	[0.148,0.504]	1.72
**PDPN**	*P*= 0.0002 (PCR)	[0.096,0.254]	2.08
**VEGFR-3**	*P*<0.0001 (PCR)	[0.370,0.686]	3.14
**LYVE-1**	*P*<0.0001 (IHC)	[0.112,0.251]	2.46
**PDPN**	*P*=0.0016 (IHC)	[0.100,0.364]	1.65
**VEGFR-3**	*P*=0.0023 (IHC)	[0.088,0.344]	1.58
**PROX-1**	*P*=0.0004 (IF)	[2.626,7.545]	1.94

2. Mouse data

In the histopathological quantitative analysis of this study, all experimental mouses were included in the analysis (no samples were excluded; n=5/group). However, in the histological fluorescence quantitative analysis, some samples were excluded due to technical reasons. Exclusion criteria included irreversible damage during staining or mounting, or unreliable data due to excessively high fluorescence signal background noise. Consequently, data from 2 mouses each at weeks 1, 3, and 6 post-surgery were excluded in the Sham and IDD groups.

Table S5. Statistical analysis data for each IDD group (mean ± SD)

Index/Week	1W	3W	6W
**Histological Score**	4.4 ± 0.55	6.2 ± 0.84	8.2 ± 1.30
**LYVE-1**	3.074 ± 0.311	4.899 ± 0.724	5.974 ± 0.199
**PDPN**	4.300 ± 0.106	5.120 ± 0.204	6.826 ± 0.642
**PROX-1**	3.429 ± 0.264	4.295 ± 0.249	6.941 ± 0.508
**MMP-13**	4.917 ± 0.418	6.392 ± 0.382	7.969 ± 0.671
**COL II**	7.864 ± 0.779	5.771 ± 0.517	4.067 ± 0.761

Table S6. Effect sizes of each factor (Partial η^2)

Index/Week	Group factors	Time factors	Interaction factors
**Histological Score**	0.87	0.60	0.54
**LYVE-1**	0.96	0.84	0.80
**PDPN**	0.92	0.66	0.41
**PROX-1**	0.93	0.89	0.81
**MMP-13**	0.94	0.70	0.60
**COL II**	0.80	0.65	0.44

Table S7. Statistical Analysis of the Sham Group and the IDD Group

**Week/Index**		**Histological Score**	**LYVE-1**	**PDPN**	**PROX-1**	**MMP-13**	**COL II**
**1W VS. 1W**	**P-value**	*P*=0.0027	*P*=0.0253	*P*=0.0007	*P*=0.0030	*P*=0.0008	*P*=0.2094
	**95% CI of diff**	[0.578,3.022]	[0.133,2.124]	[1.128,3.715]	[0.472,2.166]	[1.035,3.533]	/
	**Cohen's d**	3.28	4.98	7.39	2.63	5.34	/
**3W VS. 3W**	**P-value**	*P*<0.0001	*P*<0.0001	*P*=0.0001	*P*=0.0030	*P*<0.0001	*P*=0.0060
	**95% CI of diff**	[2.178,4.622]	[1.769,3.760]	[1.685,4.272]	[0.870,2.564]	[2.203,4.701]	[0.805,4.652]
	**Cohen's d**	5.07	5.48	7.68	9.47	5.24	3.55
**6W VS. 6W**	**P-value**	*P*<0.0001	*P*<0.0001	*P*<0.0001	*P*<0.0001	*P*<0.0001	*P*=0.0001
	**95% CI of diff**	[4.178,6.622]	[3.150,5.141]	[3.019,5.606]	[3.375,5.069]	[3.731,6.229]	[2.461,6.308]
	**Cohen's d**	5.54	18.39	5.07	11.40	9.13	5.76

Table S8. Statistical Analysis of the IDD Group

Week/Index		Histological Score	LYVE-1	PDPN	PROX-1	MMP-13	COL II
**1W VS. 3W**	**P-value**	P=0.0025	P=0.0007	P=0.2252	P=0.0374	P=0.0170	P=0.0270
	**95% CI of diff**	[0.611,2.989]	[1.082,2.568]	/	[0.050,1.682]	[0.272,2.678]	[0.240,3.945]
	**Cohen's d**	2.55	3.32	/	3.40	3.65	3.19
**1W VS. 6W**	**P-value**	P<0.0001	P<0.0001	P=0.0004	P<0.0001	P<0.0001	P=0.0004
	**95% CI of diff**	[2.611,4.989]	[2.158,3.643]	[1.280,3.771]	[2.696,4.328]	[1.849,4.255]	[1.945,5.649]
	**Cohen's d**	3.80	10.90	5.45	8.55	5.46	4.89
**3W VS. 6W**	**P-value**	P=0.0009	P=0.0053	P=0.0086	P<0.0001	P=0.0113	P=0.0724
	**95% CI of diff**	[0.811,3.189]	[0.333,1.818[	[0.460,2.951]	[1.830,3.462]	[0.3741,2.780]	/
	**Cohen's d**	1.83	2.07	3.58	6.50	2.89	/
